# What parents know and want to learn about healthy eating and body image in preschool children: a triangulated qualitative study with parents and Early Childhood Professionals

**DOI:** 10.1186/s12889-015-1865-4

**Published:** 2015-07-02

**Authors:** Laura M. Hart, Stephanie R. Damiano, Chelsea Cornell, Susan J. Paxton

**Affiliations:** La Trobe University, Melbourne, Australia

## Abstract

**Background:**

Interventions for parents to encourage healthy eating in children often do not address parental feeding practices and body image development.

**Methods:**

The current study investigated what parents (of children aged 1–6 years) understand about child healthy eating and body image, and what they would like in future interventions, by using structured focus groups with parents, and individual interviews with Early Childhood Professionals. Forty three parents (*M*_*age*_ = 36.95 years, 93 % female, 79 % university degree) participated across 9 focus groups. Eleven Early Childhood Professionals (*M*_*age*_ = 51.04, 100 % female, 64 % university degree, 64 % Maternal and Child Health Nurses, 36 % Childcare Centre Directors) completed individual telephone interviews.

**Results:**

Parents described healthy eating as a variety, balance, and range of foods as well as limiting certain foods, such as the intake of sugar, salt, and processed foods. Most often parents defined child body image as a child’s physical appearance and did not mention thoughts and feelings related to appearance or body experiences. Body image was most commonly considered a problem in early adolescence and often not an issue of relevance in early childhood. Parents appeared knowledgeable about nutrition and accessed information about healthy eating across a range of resources though rarely accessed information about child body image. They desired more practical information about how to avoid encouraging negative body image when promoting healthy eating. Professionals’ responses confirmed these findings.

**Conclusions:**

Results suggest future interventions need to stress the important role positive body image plays in encouraging healthy attitudes to food and weight management, and the benefits positive body image can have on the health and mental health of preschool children.

**Electronic supplementary material:**

The online version of this article (doi:10.1186/s12889-015-1865-4) contains supplementary material, which is available to authorized users.

## Background

Children’s diets can be influenced by a range of factors, including education environments (e.g., kindergarten or childcare) and media [1]. Parents however, not only influence these other factors, they also create the environment within which food is sourced, prepared, consumed, celebrated, resisted or refused [2, 3]. Parents shape the development of children’s eating behaviors in a number of ways, but particularly through parental feeding practices [4–6]; the “specific behavioral strategies parents employ to control what, how much or when their children eat” (p.4, [7]).

Some parental feeding practices are more likely to foster healthy eating patterns in children, while others are more likely to lead to unhealthy or disordered eating [8]. For example, parental feeding practices associated with the development of healthy eating include: repeated exposure to healthy and novel foods, positive reinforcement (using verbal praise) for healthy food choices, positive social modelling and monitoring of highly-palatable, low nutrient foods [9]. Parental feeding practices linked to weight gain, disordered or unhealthy eating behaviors include: restriction by either reducing child dietary intake or limiting snack food intake [10], pressure to eat [11], and non-nutritive instrumental practices, such as using food as a reward or to pacify [9]. While parents tend to use these latter strategies with positive intentions, to encourage healthy eating or protect against weight gain, they can have unintended consequences on a child’s food preferences, behavioral inhibition and self-regulation. Such feeding practices are therefore useful targets for preventive interventions aimed at improving parents’ ability to foster healthy eating and healthy weight maintenance in their children [12].

Research examining knowledge and use of national dietary guidelines suggests that parents understand quite well *what* they should be feeding their children, but there is a significant gap in understanding *how* to encourage their children to eat healthily [13–16]. A review of prevention interventions for weight-related problems in children concluded that effective interventions should be approached from a health-centred rather than a weight-centred perspective, with the parents as central agents of change [17]. However, most current interventions adopt a weight-centred approach by having a narrow focus on reducing child BMI (i.e., *weight*) as the primary outcome [16, 18], without assessing for any iatrogenic effects on child body image [10].

Although unhealthy eating patterns and overweight in children are serious public health concerns, the development of body dissatisfaction should be of equal concern. Body dissatisfaction predicts the development of disordered eating (i.e., fad dieting behaviours, binge eating, and using food for emotional regulation), higher Body Mass Index (BMI), less physical activity, poorer dietary quality, low self-esteem, depressive symptoms and clinical eating disorders [19–21]. Body dissatisfaction has also been found to mediate the relationships between BMI and depression [22, 23], and between overweight/obesity in teenagers and their chronic disease related health practices, such as smoking [24]. Given its negative effects on psychological resilience and health behaviours, body dissatisfaction has been called a public health imperative in its own right [25, 26].

Research on the development of body dissatisfaction in children has also focussed on the role of parental feeding practices and parental eating behaviors [27, 28]. For example, girls whose parents restrict snack foods are more likely to feel bad about themselves after consuming the restricted foods [29] and to have lower perceived physical ability [30]. Maternal dieting has also been shown to be associated with lower body satisfaction in daughters [31]. Furthermore, desire for thinness and awareness of dieting has been found to emerge in girls as young as 5–6 years [32, 33], suggesting early childhood is a critical period for intervention.

A recent systematic review of interventions for parents aiming to prevent body dissatisfaction or eating disorders, found that no interventions or evaluations existed for parents of children aged under six years [18]. The review reported that instead, researchers tend to focus on school-based interventions, often with little attention paid to the role of parents. Furthermore, studies were plagued by low sample sizes and difficulty engaging parents in prevention interventions, prompting calls for research to provide a greater understanding of what parents need and are motivated by.

For researchers to effectively engage parents in prevention programs that develop healthy eating *and* positive body image in children, we first need to understand what parents already know and want to gain from their participation. As a formative step, prior to developing an intervention to educate parents about feeding practices that promote healthy eating and positive body image in preschoolers, this research aimed to investigate what parents understand about healthy eating and body image. A focus group design was used to facilitate conversations among groups of parents. In addition, individual interviews were conducted with Early Childhood Professionals regarding their perceptions of parents’ needs. Although previous qualitative research has investigated parents’ knowledge and opinions on healthy eating and nutrition in children (e.g., [34, 35]), we believe this study is the first to investigate how parents perceive the issue of child body image and what they would like to see incorporated into future interventions that aim to improve body satisfaction and healthy eating patterns in children, and to triangulate this with the views of Early Childhood Professionals.

## Methods

### Participants

#### Parents

Parents of children attending kindergartens and childcare centres in the inner northern regions of Melbourne, Australia, were invited to participate through advertisements describing a study about promoting healthy body image and eating behaviors in young children. Directors of 34 kindergartens or childcare centres were invited by phone to advertise the study or provide an area for conducting a parent focus group. Of those contacted, nine agreed to advertise the study only (26 %) and an additional 14 agreed to advertise and host a focus group (41 %). Parent participants were required to be over the age of 18, have a child between the ages of one and six years, and to have a good comprehension of English.

Nine focus groups of between three and seven participants were conducted with a total of 43 parents. Parents were aged between 27–46 years (*M* = 36.95) and 93 % were female. The majority were married (79 %), had completed an undergraduate or postgraduate University degree (79 %), and worked part-time (51 %). The majority of parents were born in Australia (70 %), with the remainder reporting emigrating from Bangladesh, England, India, Indonesia, Iran, New Zealand, South Africa, Thailand, Ukraine and Vietnam. Ninety-five percent of parents had either one or two children aged between one and six years, with the remaining 5 % having three children. A small percentage of parents also had children over the age of six (16 %) and under the age of one (12 %).

#### Professionals

Two types of Early Childhood Professionals were invited to complete individual telephone interviews; Directors of childcare centres or kindergartens and Maternal and Child Health (MCH) nurses. Directors were included because of their role in providing resources to parents and their children, and in setting curriculum and nutrition policies in their centres. MCH nurses are publically funded specialized nursing staff who are paired with families after the birth of a child. The nurses complete scheduled child health check-ups in a community setting, and provide standardized government information on health and development [36]. Over 90 % of mothers attend at least one MCH nurse check-up with their child, making these services a rich source of information about parental behaviors and anxieties relating to healthy eating and body image [37]. MCH nurses were recruited through five local government councils of inner northern Melbourne.

Of the eleven Early Childhood Professionals interviewed, seven were MCH nurses and four were childcare centre Directors. They ranged in age from 28–59 years (*M* = 51.04), and all were female, born in Australia and had English as a first language. Six Professionals had postgraduate university training; one had undergraduate training, three had a diploma, and another had a high school certificate. Five Professionals had been working in the Early Childhood sector for over 10 years, one for 7–8 years, one for 5–6 years, three for 3–4 years and one for 1–2 years. Each of the MCH nurses worked part-time, whereas each of the Directors were employed full-time. All MCH nurses and three of the Directors were parents themselves, with between one and four children each. The children’s ages ranged between 1 and 37 years (*M* = 17.65 years).

### Data collection

Structured focus groups were conducted with sets of parents and repeated until saturation was reached and data collection no longer yielded novel results. For all groups a schedule of 11 questions was used (see Additional file 1). This centred around two themes; what parents currently know and use, and what parents would like in a new resource. The first three questions were designed to elicit information about what parents understood by the terms healthy eating and body image, and when they thought children developed a sense of their body image. The following three questions asked parents what resources they currently use to learn about healthy eating and about body image, what they like and do not like about these resources, and what they believe are the current gaps in information for parents. The remaining four questions asked parents about what would help increase their confidence in promoting healthy eating and body image in their children, what they would like to see in new information resources and what format they would most use and benefit from.

To triangulate parent data, structured individual phone interviews were conducted with Early Childhood Professionals, as logistical constraints made gathering Professionals to conduct focus group sessions, unfeasible. Again, an interview schedule was used (see Additional file 1), that was designed to elicit information about the two themes of what parents know and use, and what they need in a new resource, covering both topics of healthy eating and body image.

### Procedure

Approval to conduct this research was obtained from the La Trobe University and the Victorian State Government Department of Education and Early Childhood Development, Human Research Ethics Committees. All participants were remunerated with a $20 shopping voucher. Focus groups and interviews were conducted by research staff with postgraduate training in psychology (LMH, SRD, CC), in accordance with the schedules described above.

### Focus groups

Prior to the focus group commencing, participants were briefed on research aims and rationale, provided with written information in plain English, and given time to ask the researchers questions, before completing written informed consent forms. After consent was given, participants were asked to complete a short questionnaire about demographic characteristics, then the scheduled focus group questions commenced. One research staff member asked the group a question, then facilitated group discussion until responses had been exhausted, before moving on to the next question. All sessions were audio recorded and were completed in approximately an hour.

An essential component of assessing focus group data is examining the extent to which responses have arisen through conformance or censoring [38]. As such, our procedure involved two research staff, one facilitating group discussion, and another typing responses in a structured template on a laptop. After the 11 questions, the two research staff created a summary of the discussion by listing each novel response to each question using the structured response template, while participants had a break. This summary was then written on poster paper and presented to the group prior to session conclusion. Participants were encouraged to highlight any gaps or errors in the data recorded. This process usually prompted further discussion about how the group wanted its responses recorded by the research team. This process allowed participants to shape the interpretation of their own data and ensured that individual views were noted or consensus was achieved.

A thematic analysis of the resulting summaries was independently conducted by two members of the research team, in a two-step process. First, each of the nine focus group summaries were scanned for frequently occurring words or phrases and response frequencies were calculated using a codebook that was developed for extracting frequency data (available upon request). Second, a content analysis involving inductive category development [39] was used to create themes that grouped words or phrases about a similar topic (i.e., the theme *A variety, balance and range of foods* was designed to capture the similar parent responses - “vegetables”, “fruit”, “sweets/sometimes foods”, “variety” and “balance of food/nutrients” – to the question *What does ‘healthy eating’ in children mean to you?*)*.* When developing themes, more weight was given to higher frequency responses. The outcome was a series of themes capturing parent responses to each question. These methods have been used in previous focus group research [40].

### Individual interviews

One researcher conducted individual interviews with Early Childhood Professionals over the phone. Prior to their interview, participants were emailed a weblink to an online version of the project information statement, written informed consent form, and demographic questions. Interview responses were recorded in an electronic response template designed to capture ideas generated under each question. Interviews were completed in approximately 30 min. The same process for assessing response frequency and inductive category development was used.

## Results

### Healthy eating in children

When asked *What does ‘healthy eating’ in children mean to you?*, parents described healthy eating as a variety, balance, and range of foods (see Table 1). The importance of limiting certain foods, such as the intake of sugar, salt, and processed foods was also frequently mentioned.

**Table 1:** Parent responses to the question “What does ‘healthy eating’ in children mean to you?”

Ideas mentioned by parents	Number of groups mentioning this response	Theme developed
Vegetables	9	A variety, balance and range of foods
Fruit	9	
Sweets/sometimes foods	9	
Variety	9	
Balance of food/nutrients	7	
Following food pyramid	4	
Dairy	3	
Meat	3	
Carb/wholegrains	2	
Fresh juice	1	
Reduced sugar	4	Limiting certain foods
Homemade	3	
Reduced salt	3	
No junk/fast food/soft drinks	3	
Limited processing	2	
No preservatives	1	
Fussy eating/food refusal	7	Problems parents face in encouraging healthy eating in children
Time poor re: meal plans & food preparation	5	
Extended family	5	
Perception that healthy food is expensive	1	
Lack of social support/advice	1	
Good attitude with eating	2	Importance of healthy eating habits
Regular meals/intervals	1	
Good eating habits	1	

Although parents were not questioned about specific feeding practices, as this has been the focus of previous research [41], and the aim of the current study was to gather information about parents’ knowledge of healthy eating, body image and how they use educational resources. Some parents did however, spontaneously report using negative feeding practices, such as restriction and overt control. This was particularly evident when parents discussed the problems they faced in encouraging healthy eating, which included: managing fussy eating, finding the time to prepare healthy meals, and coping with the influence of the child’s extended family and cultural background. For example, some parents whose own parents were immigrants to Australia, reported being frustrated with the ‘old culture’, which defined healthy eating in a different way to mainstream Australian culture. One parent said *“Western style is letting baby choose their food. In my culture we would put fruit/food in front of them and chase them around the house to get the food in…[but I] want to follow a different attitude and let the baby decide.”*

Of those parents who used strategies to encourage healthy eating, the following were mentioned: encouraging a good attitude to healthy eating (e.g., not labeling food as good or bad, not using food as a reward, being a good role model by engaging in healthy eating in front of the child), the importance of regular family meal times (e.g., eating at the table and limiting television viewing), and encouraging good eating habits (e.g., making food appealing, involving the child in food preparation, dealing with resistance by persisting with presentation of novel foods, or not making a fuss if the child refuses a food). Although developing patterns or attitudes to food that would enable children to make their own healthy choices were mentioned by a small number of parents in some groups, parent discussions focused more heavily on encouraging a healthy diet (i.e., good nutrition) in their children.

When asked what resources parents were accessing for information about healthy eating, some parents reported not having accessed any. Parents who did not access resources reported not doing so because they felt their knowledge or informal support networks were already sufficient. For those who did, the following resources were reported: printed resources, such as books, recipes, research articles, health magazines, and booklets; internet based resources, such as websites and online parenting forums or blogs; professional support, such as their MCH nurse, dietitians, and childcare staff; an informal support network, for example, mother’s groups, family, friends, and other parents; and personal knowledge, including their own common sense, personal experiences, or cultural traditions.

Early Childhood Professionals viewed healthy eating very similarly to parents. They defined it as including a variety and balance of foods, as well as limiting certain foods. However, Early Childhood Professionals discussed healthy eating behaviors and habits much more often than parents; nearly half highlighted the importance of developing healthy eating behaviors in children, including empowering the child to choose which, and how much, nutritious foods to eat. For example, one professional said healthy eating is also when the *“Child feeds themselves and gets to choose (reasonably) the quantity and which particular foods they want to eat…[when children are] self-empowered eaters”.* Professionals also reported that common parent concerns were fussy eating, children’s portion sizes and not eating enough variety. MCH nurses specifically noted parental concerns around under-feeding or over-feeding their child. Directors also talked about parents being concerned that their child does not eat the same variety of food at home as at the childcare center. Many Early Childhood Professionals noted that parents typically become concerned about their child’s eating behaviors from birth, while others reported concerns arising when the child is six months old, when solids are often introduced. In line with parents’ reports, Early Childhood Professionals most frequently reported suggesting printed resources to parents, such as books, recipes, and government guidelines and initiatives, though some made reference to internet based resources as well.

### Body image in children

Not all parents could provide a definition for child body image, but when they did, parents most frequently suggested a child’s body image equated to their body size, appearance, or physical capabilities (see Table 2). For example, one parent said a good body image was *“As long as they are within the normal range…not looking too rotund and… the ribs aren’t showing so much…”.* Parents also often defined body image as what their child’s body can do (e.g., having strength) and the way their child looks, with only a few parents using the academic definition of body image as a child’s thoughts and feelings about their body and appearance. Occasionally parents reported that if a child eats healthy food, they will have a healthy body size, so the child’s body image would not be a problem. In general, parents discussed body image as negative or problematic (i.e., thought of body image as equivalent to body dissatisfaction), and largely relevant to children who were overweight or outside of the ‘norm’ (i.e., shorter, fatter, different to others). Most parents suggested that problems with body image affected girls more often than boys. Some parents endorsed the thin ideal by suggesting that if children were thinner they were more likely to be successful, so feeling bad about being bigger was not a negative thing.

**Table 2:** Summary of parent responses to the question “What does ‘body image’ mean to you”?

Ideas mentioned by parents	Number of groups mentioning	Theme developed
Equates to body size/weight/appearance	7	Body image is about a child’s body size, appearance, or physical capabilities
More about what body can do (e.g., strong/energetic)	6	
If child is healthy, body image shouldn’t be a problem	2	
Relates BI/size to food	2	
Not about looking a certain way	1	
Healthy eating & phys act = looking healthy = body image	1	
How child feels about self/body	7	Body image is a child’s perception and emotions related to their body
Problematic e.g., ‘demons’	3	Body Image is a problem for children
Positive & negative	1	
Good body image is not expressing concern about the body	1	
Girls more than boys	9	Negative body image mostly affects girls
Can affect anyone	1	
Being thin increases your chance of success	2	Endorsing the thin-ideal

Given the uncertainty among participants about the meaning of body image, before asking parents any further questions the facilitator clarified that the researchers understood children’s body image as how the children thought and felt about their bodies, not just about how they looked. In addition, the facilitator explained that these thoughts and feelings could be positive or negative.

When asked about when children develop a sense of their own body image, parents most often reported that this occurred around three to four years of age. It was often noted that a child’s body image developed at a younger age in girls than in boys. Parents also frequently reported that children develop body dissatisfaction, or experience difficulty with body image, later on in childhood or adolescence, especially around puberty.

Although not specifically questioned on how parents become aware that their child was developing a body image, parents spontaneously reported the following behaviors as cues: through preferences in clothing and dressing themselves, wanting to look like another or to look a certain way, making comments about their own body (e.g., ‘I’m fat’) or making comments or teasing others (e.g., ‘you shouldn’t wear that because you’re fat’), copying what they see or hear from others (e.g., developing an interest in fashion or what their friends are wearing) and being conscious of their appearance, but not necessarily in a negative way.

Parents also often spontaneously discussed what influenced children’s body image development. Most parents mentioned socio-cultural factors, for example exposure to unrealistic images in the media, in children’s story books, and toys such as Barbie (see Table 3). Peers were also mentioned, especially in relation to children making body comparisons with peers. Less frequently observed comments about influences on body image included: parent’s own body image (e.g., weighing themselves and commenting on their own appearance), indirect comments about appearance (e.g., general appearance comments made about, or to, other people in front of their child, and the comments others make about their own appearance in front of the child), direct comments about their child’s appearance (e.g., the parent’s and other’s comments about how the child looks), siblings (e.g., one parent said “*I catch my 8 year old standing at the mirror checking herself out. Then I catch the 5 year old do[ing] exactly the same*”), and the child’s body size (e.g., being taller or heavier than the ‘norm’).

**Table 3:** Summary of parent ideas about influences on child body image

Ideas mentioned by parents	Number of groups mentioning	Theme developed
Child’s body size (e.g., height or weight)	3	Child’s body size
Parent’s body image (e.g., own body image impacting child)	5	Parent body image
Parent weight/appearance behaviors (e.g., weighing self)	1	
Parent comments on own appearance	2	
Language (e.g., Fat)	1	Indirect comments
Parent comments in general (e.g., Use of word fat)	2	
Parent comments about others around child	2	
Parent comments to others around child	1	
Others comments/behaviors about child appearance	2	
Parent comments to the child about their appearance (direct)	1	Direct comments
Others commenting on child	6	
Sibling (e.g., imitating older)	3	Sibling
Socio-cultural influences in general	4	Socio-cultural factors
Media	7	
Children’s stories/books	6	
Barbie	2	
Activity (e.g., dancing)	1	
Role modelling (e.g., child influenced by someone they admire)	1	
Peers who are aware of their body image	6	Peers
Start socialising with other children	1	
Starting kinder	3	
Starting school	2	

When asked about what resources parents used to learn about child body image, most participants reported not having looked for any information. For those parents who had looked, they reported a general lack of resources aimed at this age group or did not know where to look. Other parents reported using their personal knowledge or experience related to body image. Although many parents reported not knowing how to promote a positive body image in their children, some strategies they mentioned using included: placing an emphasis on health rather than appearance (e.g., discussing the body’s energy, strength, what it can do, and avoiding associating food with body size), talking about food in the context of health rather than weight (e.g., one parent said *“..... if they’re having bacon and eggs and they cut away the fat or they see their father cutting away the fat, I tell them it has do with how your arteries behave when you put too much fat in. It’s nothing to do with having a large exterior…”*), promoting body acceptance (e.g., encouraging their child to value different body shapes and sizes, and encouraging their child to be critical of what they see in the media), and avoiding ‘fat talk’ (e.g.*,* not using the word ‘fat’ and being conscious of appearance related comments about their child and others).

Most Early Childhood Professionals identified body image as the child’s thoughts and feelings of their own body*.* A small number of Early Childhood Professionals, however, did define body image as how a person looks or their body size. All but one Early Childhood Professional reported that children develop a sense of their body image at a young age, from birth to five years old, with three to four years old being reported most frequently.

Early Childhood Professionals also often discussed the influences on child body image. They noted: family (e.g., parents and siblings who make comments about, or model the importance of, appearance); peers (e.g., through teasing or appearance-based comments if a child is different to the ‘normal’ body size); and other socio-cultural factors (e.g., through the media, clothing, and culture).

Many Early Childhood Professionals reported that body image was not yet an issue for parents, but that children’s body weight was cited as a common concern (e.g., one Maternal and Child Health nurse reported that *“Around 12mths of age, parents often say ‘I don’t want to give my child too much because don’t want him to get fat’”*). It should be noted that the final check-up performed by MCH nurses is when a child turns three and a half, and children in Victoria start primary school, and therefore exit childcare, at age 5. So Professionals were reporting on their experience of parents who had children at or under the age of 5 years. Professionals noted that when parents were concerned about body image, it was because they were worried about their child being overweight and getting teased, or their child looking underweight, even when they were in the healthy weight range. Professionals also reported that parents’ own experiences with their weight influenced their concerns about their child’s weight, along with pressure from other family members for their child to be big. The majority of Professionals had not provided any body image resources to parents, yet they noted that this was because of the young age of the children they saw and the lack of parents requesting such a resource.

### What parents want from a healthy eating resource

When parents were asked what they would like to see in a new resource they reported wanting a resource to include (see Table 4): healthy eating strategies (e.g., dealing with fussy eating with minimal stress, how to get children to eat a wide variety of food), information about how to encourage good nutrition (e.g., making good nutrition a priority in the child’s life, understanding portion sizes for children, positive and negatives of limiting food choices), and recipe and meal ideas.

**Table 4:** Content parents would like in a new resource for parents to encourage healthy eating and body image in children

Ideas mentioned by parents	Number of groups mentioning	Theme developed
Strategies for encouraging healthy eating, which create minimal stress and overcome fussy eating	9	Healthy eating strategies
Hiding food ideas	1	
How to get variety in food	2	
How to promote healthy habits around food (i.e., in the same way we have information about how to promote healthy sleep habits in children)	5	
Importance of healthy eating (i.e., how to make it a priority)	1	Food & Nutrition
Information on portion sizes	2	
Nutrition information and how it relates to physical activity	3	
Food pros/cons/substitutes (e.g., skim vs full cream milk)	4	
Drinking water guidelines	1	
Health risks/implications associated with eating ‘unhealthy’ foods	3	
Ideas for healthy lunchbox	2	Recipe/Meal ideas
Recipes/menus for healthy family meals	7	
Strategies for parent modelling healthy eating & body image	2	Parent modelling
How to encourage positive body image (e.g., building child self-confidence/resilience)	8	Strategies for promoting positive body image
How to talk about food/body (especially knowing what not to say/how to avoid things that will make it bad)	4	
How to teach child to accept differences in bodies	2	
How to deal with external influences on body image (e.g., other adults, children’s parties)	6	Dealing with external influences on body image
Media literacy strategies	2	
What to do if child is over/underweight/not growing enough	1	Early detection/intervention strategies
How to identify body image influences/risks & what to do about it	4	

The majority of Early Childhood Professionals suggested a stronger focus on educating parents about strategies for encouraging healthy eating in children (e.g., importance of healthy eating at a young age, modelling healthy eating, how to address fussy eating, and not using food as a reward, and the long term consequences of unhealthy eating in children). They also felt that it was important for parents to help children develop their own healthy eating habits by allowing children to have autonomy over their eating (i.e., parents provide healthy food choices and the child is then allowed to choose what they want to eat and how much). For example, when asked what kind of information a new education package should include, one professional said it is *“Important to communicate that the child [should be] allowed to feed themselves as soon as possible. Allowing the child to choose the volume of food they need and the variety of foods they want ([though] it will change day to day and that’s ok), is part of the child being resilient.”* In line with the request from parents about recipes, some Professionals suggested having healthy food ideas for children (e.g., school lunchbox ideas), along with providing information about quantities and portion sizes for children.

### What parents want from a body image resource

As shown in Table 4, parents reported wanting a body image resource to include: strategies for promoting positive body image in their child (e.g., how to talk about food and the body in ways that won’t negatively affect their child’s body image). For example, one parent questioned *“How do I explain to a five year old that they are not fat…how do they even know that word?”*. Parents also wanted information on how to deal with external influences (including messages from the media), and on early detection and intervention strategies (i.e., how to identify problems with their child’s body image, risk factors, influences, and what parents can do about them).

Many Professionals concurred with parents, suggesting that a resource that gives parents guidance on how to talk about body image with their child, and when parents should be concerned about their child’s body image, would be extremely useful. The Professionals also mentioned the importance of highlighting how parental modelling, genetics and sociocultural factors all influence body shape and size. MCH nurses in particular suggested that having information about physical changes that occur as a child gets older, how these changes relate to children’s food requirements, and how children will naturally develop different body sizes, would be helpful for parents.

### Delivery and format of a new resource

As shown in Table 5, parents frequently reported wanting something quick, simple, and easy to read, with options provided for further reading. Participants mentioned a preference for dot points using clear and plain language, following a factsheet format. A quick-guide ‘do’s and don’t’s’ checklist was mentioned by some, though the idea of providing realistic guidelines, with tips and stories from other parents, was highly endorsed. Parents also wanted the resource to be easily accessible (e.g., across multiple formats, such as internet and in print) and appropriate for all families (e.g., across culture and education levels). Parents also wanted information to be evidence-based with acknowledgments of the evidence source. One parent reported wanting *“Dot points…of evidence-based research....to quickly get information and then read on if you want, with resources at the bottom”.* Parents also mentioned a preference for an educational resource they could use with their child, such as a book, toy, or game activity.

**Table 5:** Format parents would like for a new resource for parents to encourage healthy eating and body image in children

Ideas mentioned by parents	Number of groups mentioning	Theme developed
Something we can come back to	5	Ongoing resource
Book	4	Paper resource
Booklet	7	
Downloadable booklet	2	
paper	1	
Folder for fact sheets	1	
Website	8	Electronic resources
Online forum	3	
Email	3	
Facebook	1	
Blogs	1	
Electronic resource	1	
Flyers to advertise website	3	
Seminar/info night	5	Seminars
- with follow-up session	1	
DVD/video	3	Visual
Visual/graphs/pics	3	
Multiple formats	1	Other
Multilingual	1	
Simple/easy/quick (parents are busy)	7	Quick/Brief
Brief, with options for further reading	7	
Target to all parents & children (e.g., Genders, cultures, SES, rural)	8	Be accessible to wide range of parents
Accessible	4	
Realistic/applicable guidelines/options	9	Realistic guidelines
Dos & don’ts (promote health, avoid damage)	3	
Checklist	1	
Evidence based research	8	Evidence base
Reasons for strategies (why it will work)	2	
Acknowledge source of info	1	
Resource for child (e.g., book/toy/activity/app)	7	Child resource
Tip sheets/fact sheets/dot points (but not too many)	6	Tip sheets/dot points
Clear/plain language	1	
Parent tips/stories - yes	5	Parent stories
- not helpful (do not include)	2	
Resource for instructors e.g., Dance/sport	1	Supplement for non-parents

Parents reported wanting to access a resource about healthy eating and body image when it is developmentally relevant to their child’s age. However, the age at which healthy eating was deemed relevant was very young (e.g., between birth and six months), whereas the age of relevance for body image was considered much older (e.g., when the child becomes concerned with their body, in late childhood or around puberty). Although parents did not want to introduce the concept of body image to their child too early, they did nevertheless want to be aware of risk factors and strategies to help prevent body dissatisfaction. One recommendation to overcome this conflict in timing was to provide a resource that parents could revisit when relevant to their individual circumstance. Others reported wanting resources for healthy eating and body image to be provided separately; a healthy eating resource when their child was around six months old, and a body image resource once their child was three years.

Consistent with the parents’ views, the majority of Professionals thought the internet would be the best way to deliver information to parents, including having an online forum where parents could share their experiences. Having seminars or visual presentations, ideas for further reading, and printed resources (e.g., factsheets, pamphlets, booklets, parent newsletters) were also recommended. Professionals suggested that the resource should be quick and easily accessible, updatable, ongoing and not tokenistic. They thought it was important to include flexible options for parents to explore. Through provision of such a resource in their centres, Professionals reported being likely to engage in assisting parents to help promote healthy eating and positive body image in their preschool children.

## Discussion

The current study used structured focus groups to gather data from parents, and individual interviews with Early Childhood Professionals to triangulate parent data, to investigate what parents understand about healthy eating and body image in children, and what they would like to learn from future public health interventions. The results suggest that parents understand the basic nutrition components of healthy eating. They want to learn how to implement dietary guidelines when children are fussy, refuse healthy food, or when they are time-poor and have limited options for making menu choices available. In other words, parents want information on positive feeding practices and strategies to avoid developing negative ones. In addition, many parents appear to know fairly little about child body image and generally only consider it relevant to parenting when it becomes an issue in later childhood, preadolescence or at the time of puberty. In agreement with current reviews of the literature (e.g., [16, 18]), our results suggest that there is a clear and current gap in information that meets the needs of parents in developing healthy eating patterns and positive body image in preschool children.

Consistent with the recommendations made by Schwartz et al. [16], parents in the current study called for more information about how to help children achieve healthy eating habits and reported frustration with brief resources that did not allow for flexibility in meeting their individual circumstances. Developing information on parenting strategies that help children to develop healthy eating patterns would be more likely to meet these multiple demands of parents, than the simple national guidelines currently available. Our results therefore concur with previous research that suggests parents understand *what* they should be feeding their children, but would like more information and guidance on *how* to achieve this.

A longitudinal study investigating the prospective relationship between parental feeding practices and child weight found that among children predisposed to obesity, elevated child weight appears to elicit restrictive parental feeding practices, which in turn produced additional weight gain [42]. The authors stressed the need for guidelines on obesity prevention that included consideration of child characteristics, such as vulnerability to obesity and current weight status, to inform parental feeding practices. In the current study, parents’ request for information about how to limit and talk about ‘unhealthy’ foods without having a negative impact on the child’s developing attitudes, importantly underlines parental awareness of how restricting and controlling practices may have negative effects. Nevertheless, parents appeared to engage in these pitfalls and desired more guidance on what strategies to employ. This finding strengthens the call for future interventions to focus on providing practical strategies that meet parents’ needs, and allow them to respond to their child’s individual circumstance, rather than relying on nutrition education alone.

In contrast to healthy eating in children, about which parents appeared fairly knowledgeable, few participants had a good understanding of child body image. Interestingly, parent conceptualizations of child body image often appeared to equate with a child’s physical health; many parents remarked that if a child was fit and healthy, then the child would have a good body image. While research suggests that there is an association between healthy weight and positive body image [20, 43], healthy weight does not necessarily protect against body dissatisfaction. For example, research has suggested that 59 % of 5–8 year olds would like their figure to be smaller [32], and that even 10 % of thin or underweight children desire to be thinner [44]. Relying on healthy weight alone is, therefore, not sufficient to protect a child against feeling dissatisfied with their appearance. Given this, it is important that parents understand that a healthy-weight child will not necessarily feel good about his or her body, and instead, parents need to encourage a positive cognitive framework in relation to their child’s body and appearance, in much the same way as many parents understand and aim to foster healthy self-esteem.

Although some parents discussed the relationship between healthy eating, healthy weight and positive body image, parents’ conception of these relationships appeared to be unidirectional; there was no mention of the idea that positive body image in children could be fostered and used to encourage healthy eating and in turn, healthy weight management [24]. Educating parents about the benefits of positive body image [20, 45, 46], would probably assist them in understanding why it is important to encourage children to have helpful thoughts and feelings about their body from preschool age, rather than waiting until later childhood when their body satisfaction may already be an issue. Clearly, our results suggest that information about the importance of fostering positive body image, irrespective of the child’s weight, and provision of strategies that guide parents to do so, are needed in future intervention resources.

Finally, the recommendations from parents about the format and dissemination of future interventions suggest that creativity and a good understanding of parental needs are required when developing resources. In particular, the suggestion that a resource be made available to parents across multiple formats, such as in printed booklets, on a website or in a face-to-face workshop, is supported by health behavior research that demonstrates that providing multiple opportunities for individuals to engage with material designed to change knowledge and behavior, improves the chances that the intended changes take place [47]. In fact, researchers wishing to design interventions to guide parents in developing healthy eating and body image in their children would be wise to use health behavior models to inform their programming, in addition to the standard approach of developing intervention content targeting reduction of established risk factors [18, 48]. Parents’ call for incorporation of an educational task or activity that they could do with their child also complements an ecological approach to prevention programming, whereby intervention content focuses on multiple sources of influence in a child’s environment [48].

Although parents often thought information about body image was only relevant in late childhood, researchers would be wise to provide an integrated intervention, describing both practical strategies for using positive parental feeding practices, with information about how to promote a positive body image, to parents early in a child’s life. This would provide two benefits. First, given that parents and Early Childhood Professionals agreed that parents desire information about healthy eating as early as six-months of age, researchers could take advantage of parent willingness to engage at this early stage. Second, developing parents’ understanding of the important relationships between positive body image, healthy eating and healthy weight management, would assist them in promoting the healthy eating patterns they desire for their children, as well as equip them with appropriate skills required for promoting healthy body image, even in children as young as 2–6 years.

A limitation of the current research is that results were obtained from relatively well educated parents, a very large majority of whom were mothers, and from Early Childhood Professionals living and working in the northern regions of Melbourne, Australia. It is possible that if conducted elsewhere, the gathered data may have been different, particularly where education levels, cultural food practices and paternal engagement contrasted with the norms of the area sampled. Previous research has shown that level of education, cultural background and maternal/paternal roles can all influence perceptions of child eating and parental feeding practices [5, 49, 50]. The generalizability of these data are therefore limited to other samples of similar composition. However, given that very little research to date has focused on assessing the needs of parents to inform future prevention interventions [18], we believe these results provide an important though initial insight to what parents know and want to learn about healthy eating and body image in preschool children.

## Conclusions

Parents of children aged one to six years appear to be engaged in learning about healthy eating in preschoolers. They are knowledgeable about basic nutrition and want to encourage their children to eat healthily, though often lack practical strategies in how to achieve this. In contrast, most parents appear to know little about the development of child body image, generally are unaware of the positive relationship between body satisfaction and health behaviors, and believe body dissatisfaction only becomes relevant to parenting in later childhood. Our results suggest that there is a clear and current gap in information that meets the needs of parents. If future public health resources, designed to prevent eating, weight and body image problems in young children, are to be effective in meeting parents’ needs, they should include practical parenting strategies presented across a range of formats and in accessible language and style. Importantly, they must also emphasize the important role parents can play in developing both healthy eating and positive body image in early childhood.

## Additional file

Additional file 1:
**Focus Group and Interview Schedules.**

## Abbreviation

MCH Nurses: Maternal and Child Health Nurses
