# Supplementary material for: What parents know and want to learn about healthy eating and body image in preschool children: a triangulated qualitative study with parents and Early Childhood Professionals
Source: BMC Public Health. 2015 Jul 2;15:596. doi: 10.1186/s12889-015-1865-4 (PMC4487845; doi:10.1186/s12889-015-1865-4)
Supplement: Additional file 1: — Focus Group and Interview Schedules. [file 12889_2015_1865_MOESM1_ESM.docx]

**Additional file 1: Focus Group and Interview Schedules**

## Focus Group Schedule

**Questions to guide focus group discussion with parents**

1. What does ‘healthy eating’ in children mean to you? (We would like to focus on children aged 1-6 throughout this session).
2. What about ‘body image’?
3. *(Provide definition of body image: the thoughts and feelings about the body and appearance)* When do you think children develop a sense of their own body image?
4. What resources designed for parents that provide information on how to encourage healthy eating or physical activity in children, are you aware of/do you use?
5. What resources designed for parents that provide information on how to encourage positive body image in children, are you aware of/do you use?
6. What don’t you like about these resources, or about how parenting information is currently delivered?
7. What gaps do you think there are in current resources/information for parents? What would you like to see developed to help you promote healthy eating and positive body image, in your child?
8. Is there anything else that you feel would help increase your confidence in promoting healthy eating, physical activity and positive body image in your child?
9. If new information/resources for parents were developed, when do you think would be the best time for parents to receive this information (e.g., as soon as you become a parent? As the child gets older? At what age?)
10. If new information/resources for parents were developed, what would be the best ? (e.g., information sheets, website, training course, consulting a professional)
    1. Would you use a website with information about how to promote healthy body image, eating and physical activity in children?
    2. Would you use booklets?
11. What would this package need to include to help you access and use the information (e.g., clear language, stories from other parents, videos, professional opinion, evidence based research??)

**Interview Schedule**

**Interview Questions for Early Childhood Professionals**

**Background**

1. What does “healthy eating” in children mean to you? (We would like to focus on children aged 1-6 throughout this session).
2. What about “body image”?
3. *(Provide definition of body image: the thoughts and feelings about the body and appearance)* When do you think children develop a sense of their own body image?
4. What resources designed for parents that provide information on healthy eating or physical activity in children, are you aware of/ given or suggested to parents? (e.g., websites, brochures, books, courses, organisations)
5. What resources for parents about body image in children?
6. Do you mention or give these resources to parents? (e.g., through conversations during appointments or handing out information sheets)

**Parental concerns**

1. What are some common concerns that parents discuss with you, about their preschoolers’:
   1. healthy eating
   2. body image
2. At what point in the child’s life do parents become more focused on or concerned about their child’s healthy eating behaviours and body image? (*interviewer note: if MCH nurses believe these emerge at different times, ask at what age each emerges*)

**Design of an education package**

1. What kind of information would you suggest we develop to address parent’s concerns about healthy eating and body image? (*interviewer note: if MCH nurses believe these should be addressed separately rather than in combination, ask how each should be addressed*)
2. How would this information best be delivered to parents?
3. What do you think is not effective when working with parents (what should we avoid)?
4. **Role of MCH nurses/centres (Questions for MCH Nurses Only)**
5. What could be done to help support you in dealing with issues relating to healthy eating and body image with parents?
6. What role do you think MCH nurses or centres could play in providing an education package to parents that aims to promote healthy eating and positive body image in children?
   1. Education/counselling on these topics during your routine sessions?
   2. Provide handouts or booklets?
   3. Provide promotional materials with links to a website or phone app?
7. What other organisations or structures do you think could be involved in providing this package to parents (e.g., kinders, child-care centres)?
8. **Role of Child Care Centres/Kindergartens (Questions for Directors Only)**
9. What could be done to help support you in dealing with issues relating to healthy eating and body image with parents?
10. What role do you think Child Care Centres/Kindergartens could play in providing an education package to parents that aims to promote healthy eating and positive body image in children?
11. Education/counselling on these topics?
12. Provide handouts or booklets?
13. Provide promotional materials with links to a website or phone app?
14. What other organisations or structures do you think could be involved in providing this package to parents?
